# Cases of Emphysema, Depending upon Different Causes

**Published:** 1831-10

**Authors:** P. Meniere


### EMPHYSEMA.

*Cases of Emphysema, depending upon different Causes.*
By P. Meniere, d.m.p. &c.*

The introduction of atmospheric air into the intermuscular and subcutaneous cellular tissue, frequently takes place from wounds which penetrate the chest. In most instances an attentive examination of the injured parts explains the cause of the emphysema: it sometimes, however, happens that the sound state of the lung, or some other circumstances, do not enable us to account for it satisfactorily. Emphysema may occur although the costal pleura has not been injured; in fact, it may arise spontaneously. The following are interesting examples of different varieties of emphysema.

Case I. Marie Tauvrai, æt. twenty-six, of a vigorous constitution, received, in a scuffle, a violent blow on the internal angle of the left eye. She suffered great pain, and fell to the ground. A few drops of blood having been discharged from the nostrils,

* Archives Générales de Médecine.

she blew her nose with some force. The expiratory effort which she made after having closed the nostrils, caused an immediate swelling of the eyelids. The same act being repeated twice or three times, considerably increased the tumefaction of these parts, and they could no longer be separated. The bleeding from the nose had entirely ceased. Under these circumstances, she was admitted into the Hôtel Dieu of Angers, on the 18th May.

Both lids of the left eye, and the adjacent parts of the cheek and forehead, as well as the upper part of the nose, were much swollen. The skin was tense, shining, and elastic; crepitation was detected by very slight pressure. The eyelids were so completely closed, that it was impossible to ascertain the state of the globe of the eye. The colour of the skin was unchanged; there was neither lachrymation nor pain. As there were some doubts as to the accuracy of the report made by the patient, she was desired to blow her nose in the presence of the physicians: the moment the nostrils were closed, and the external egress for the air was shut, the tumefaction of the eyelids increased. The experiment gave great pain. The swollen parts were covered with lint soaked in cold water.

19th. Tumefaction diminished; no change in the colour of the skin. Subcutaneous crepitation was very distinct, and the same result followed, but in a less marked manner, from closing the nostrils, and making an expiratory effort. The same application was continued. In the evening, the swelling of the eyelids was sufficiently diminished to permit an examination of the globe of the eye, which was found to be free from injury: vision unimpaired.

20th. Scarcely any tumefaction remained.

In the course of the next night, the patient, while half asleep, felt a disagreeable itching in the nose, which caused her to sneeze. She blew her nose with some violence, and the emphysematous swelling immediately reappeared, in its original size and form: great pain again came on, and in the morning the eyelids could not be separated. Upon each effort to expire when the nostrils were closed, the swelling increased, and particularly of the upper eyelid. The same treatment was adopted as before, and she left the hospital perfectly cured on the 24th.

All writers, who have described traumatic emphysema, mention that of the eyelids, and point out the rapidity of its formation, which they ascribe to the laxity of the cellular tissue of the parts: but no notice has been taken of emphysema confined to the eyelids, and depending upon a cause which has acted directly upon them.* M. de Wenzel

* Mr. Mackenzie makes the following remarks upon this subject in his "Practical Treatise on Diseases of the Eye," p. 133.

" *Emphysema of the Eyelids.* A swelling of the eyelids, produced by the presence of air in their cellular membrane, may either be part of a general speaks of emphysema as an accident which sometimes arises from the operation for cataract, and refers to several authors for a confirmation of the fact. It is clear that in the above case the air passed directly, and with facility, from the interior of the nasal fossæ into the substance of the eyelids: the blow probably separated the edges of the superior maxillary bone from the os unguis. This diastasis was no doubt accompanied by laceration of the fibrous membrane which lines the orbit, and of the cellular membrane which extends into the ethmoideal sinuses. The hemorrhage which took place from the nose indicated this laceration, without which the rapid passage of the air into the substance of the eyelid would have been impossible. The treatment was very simple, and probably all applications might have been dispensed with, as there were no serious symptoms to remove.

Case II. A man, forty-six years of age, of robust frame, was brought into the Hôtel Dieu, in a senseless state. There was profound coma, stertorous breathing, complete relaxation of all the limbs; no external injury to be detected. The jaws were powerfully convulsed, and the muscles of the neck stiff. Upon closing emphysema, arising from an injury of the organs of respiration, in which case the air, escaping from the lungs, spreads through the whole body, and accumulates chiefly where the cellular substance is loose; or it may be the consequence of a fractured frontal bone, the air passing through the frontal sinus, and through the fracture, into the eyelids. The following is an instance of the latter variety of emphysema of the eyelids: A boy, sixteen years of age, as he was going along the street, with a load, ran inadvertently against a person passing in the opposite direction; a scuffle ensued, in which he received a severe blow over the right frontal sinus. About an hour after, having occasion to blow his nose, the eyelids and parts adjacent became immediately inflated, so as completely to close the eye; and he felt the air rush, he said, into those parts. On being admitted into Guy's Hospital, under Mr. Morgan, the eyelids were much distended, and so closely approximated, that they could not be separated by any voluntary effort of the patient; the eyebrow was also puffed up, and the cellular membrane between the ear and the orbit was in the same state of emphysema. The parts were not at all painful on pressure; they yielded a crackling sensation to the touch, and were free from discoloration. The supposed seat of the fracture was at a small distance above the superciliary ridge, where a slight depression, but no crepitus, could be felt. The globe of the eye was perfectly natural. The treatment adopted was very simple: two small incisions were made through the integuments, about the eighth of an inch behind the external angle of the frontal bone, which allowed the air to escape. The swelling subsided in twenty-four hours, leaving the eye and surrounding soft parts in a perfectly healthy condition. The same plan of incision through the integuments is adopted when the eyelids are greatly distended in cases of universal emphysema. It is merely, of course, a palliative remedy; the complete removal of the disease depending on the healing-up of the injured part of the lungs or windpipe. Even in cases of fractured frontal bone, the evacuation of the diffused air is merely palliative; and till the consolidation of the bone the emphysema will be liable to return."

the nostrils so as to prevent the exit of air, respiration was suspended for about half a minute; the patient then forcibly expired, and the right upper eyelid was seen to swell a little. The mouth was closed, and the air passed entirely through the nasal fossæ: the eyelid was soon considerably tumefied, and an emphysematous crepitation was very distinct. This symptom led to a more accurate examination of the eyelid, and there was found, besides a slight abrasion of the skin, a yellowish appearance, manifesting that the part had previously suffered some contusion. M. Menière now concluded that there was probably a fracture either of the bones of the orbit or the base of the cranium, as the ethmoid or sphenoid. The air, penetrating into the cells and sinuses of these bones, had found an issue into the substance of the eyelid, when any obstruction opposed its exit by the nostrils.

The patient died the following day. It was afterwards ascertained that he had been assaulted by several men about a fortnight before, and that he had received a blow on the face from an umbrella, which had struck him senseless to the ground.

*Dissection.* A fracture of the orbit was found, with laceration of the anterior lobe of the cerebrum. The dura mater was separated from the bone around the fracture, but it was not torn. One of the bony fragments extended into the frontal sinus, and communicated with the ethmoidal cells, which contained a little liquid blood.

Here no doubt could be entertained as to the nature of the injury which had occasioned the emphysema. The principal advantage to be derived from this case is the fact, that the passage of air into the eyelids may tend to establish our diagnosis of fractures of the base of the cranium. The emphysema which occurs in the parietes of the thorax when they have been wounded, does not always depend upon the passage of air from the lungs: J. L. Petit saw a case in which there was a round wound, with lacerated edges, about three inches below the axilla, which had caused considerable emphysema of the whole of the chest on the injured side. In this instance the most careful examination proved that the wound, which had been made with a pointed stick, had not penetrated the cavity of the thorax. Incisions were made in different directions, and a deep sinus was discovered on the external surface of one of the ribs: the cure was completed in a month. The circumstances of the following case differ but little from that of Petit.

Case III. Pierre Renaud, æt. twenty, a dragoon in the 24th regiment, in a fit of intoxication, quarrelled with one of his officers. From great alarm at the consequences which might follow this insubordinate conduct, he determined to destroy himself: he placed the hilt of his sabre on the pavement, held the point towards his chest, and threw himself upon it. The weapon penetrated the interval which separates the cartilages of the sixth and seventh ribs on the right side, and slipped along the inferior edge of the great pectoral muscle to just beyond the breast. He was immediately taken from the ground: violent hemorrhage ensued, and vermilion-coloured blood was discharged by jets. The bleeding was arrested by pressing the wound with handkerchiefs.

The man was carried to the hospital five hours after the injury, in the following state: The wound was two inches and a half in length, running obliquely from below upwards; the cartilage of the seventh rib was divided through three parts of its substance; from the upper angle of the wound to the nipple, there was a slight protuberance of a round shape, and painful to the touch: upon pressing it from above downwards, a good deal of half-coagulated blood was discharged, and upon approximating the edges of the wound, to unite them by the first intention, a small quantity of arterial blood escaped, but the hemorrhage soon ceased. A moderately tight bandage was applied round the chest, and blood was taken from the arm. The patient still greatly agitated; respiration free; no pain, except when the seventh rib was pressed upon. An hour after the accident, he had bled from the nose rather freely: he had neither cough nor increased expectoration. In the evening, the parts around the wound, to the extent of three or four inches, were found to be emphysematous; pulse full and large; eyes turgid. Twelve ounces of blood were taken from the arm.

30th October, (day after the injury.) Patient slept well; tongue dry and red, which may be accounted for from the excess committed yesterday; pulse tranquil; mind easy. Emphysema has extended in the direction of the axilla; no bleeding; about one half of the wound united. In the evening, better in every respect: no further extension of emphysema.

31st. Appetite returned, and other indications of health; emphysema diminished; the surface of the lower part of the chest of a yellow colour; wound completely united.

In eight days the patient left the hospital cured.

In this instance, the presence of air in the neighbourhood of the wound did not depend upon any injury of the pleura costalis, nor of the lung. There was no symptom, in fact, which indicated the entrance of air into the cavity of the chest: the rapidity of the cure shews that the wound had not penetrated the thorax. But how is the occurrence of emphysema to be explained? It could not be attributed to decomposition of the fluids from the injury received, for no decomposition took place; and, besides, this symptom appeared too suddenly to be referred to such a cause. It must be presumed, then, that the successive movements of elevation and depression of the thorax are sufficient to give entrance to the air into the cellular tissue, when the substance of the thoracic parietes is merely wounded, and not cut through, and particularly when the introduction of air is facilitated by the thinness of the patient.* In some cases, emphysema arises so suddenly and to so great an extent, where the wound of the serous membrane covering the lung is very small, that it is difficult to conceive that lesion can be the only cause of the symptom. The following is an example of this kind.

Case IV. An insane patient, æt. fifty-six, fell upon the edge of his bed, from a height of about eight feet. In about a quarter of an hour after the accident, M. Menière saw him: he was in a state of great agitation; pulse small and hard; skin cold; lying on his back; extreme dyspnœa; the left side of the chest very painful when touched; several ribs fractured; about six square inches of the thorax emphysematous. A tight bandage was applied round the chest; free bleeding from the arm.

Four hours after the fall, all the symptoms were much increased: dyspnœa excessive; emphysema extending to the neck, and occupying the whole of the chest; abdomen also slightly emphysematous. Thirteen hours after the accident the man died.

*Dissection.* The four first ribs and the twelfth were the only ones uninjured; all the rest were fractured in their posterior half. The lung adhered forcibly to the four upper ribs. Opposite the fracture of the sixth rib was found a small opening, about two lines broad, in the pleura: the part of the lung situated opposite this opening exhibited a still narrower wound, around which the tissue of the organ was perfectly sound. There was neither interlobular emphysema nor effusion of blood into the pleural sac.

In this instance, the wound of the pleura was very small, and yet the developement of emphysema was extremely rapid. There are many facts on record which prove that the most minute divisions of the pleura give an easy passage to the air, and are followed by enormous emphysema; but this result usually occurs after a considerable lapse of time, and here it arose almost instantaneously.

Emphysema may arise from other causes than external injuries. Authors have particularly described it as occurring in the front of the neck after violent fits of breathing; powerful efforts during the act of labour; long-continued cries; any mechanical obstruction to the exit of the air by the ordinary passage, are the most frequent causes of emphysema, when no bad effects result from it. In three

* Mr. Hewson states, that wounds which penetrate the thorax, without injuring the lungs, may give admission to air from without; the air entering the wound during inspiration, and being expelled during expiration.—Editor.

women, during their first labour, which was long and difficult, both from the narrowness of the pelvis and the size of the child's head, emphysema occurred. Each of these females was thin, and of feeble health: neither of them was sensible of the accident, and neither felt any pain in the chest or trachea; the emphysematous swelling arose suddenly, and scarcely caused any inconvenience: it did not extend to the face, and was principally confined to the space between the sterno-mastoidean muscles; colour of the skin remained unchanged: it quickly dispersed, leaving no trace of its appearance, the third or fourth day after labour. No medical treatment was employed.

In one of these women the stethoscope was applied, but M. Menière detected no particular sound. The volume of the tumor was particularly increased during expiration: the physiological reason of this phenomenon has been pointed out with great precision by M. Cloquet. In a hydrophobic patient, emphysema of the neck occurred soon after the commencement of the disease, and extended behind the ears: the violence of the efforts which he constantly made, his incessant cries, and the powerful attempt at expiration, rendered his condition analogous to that of a woman in labour, and gave rise to a similar effect. M. Menière has attentively watched eight other hydrophobic patients, but no such event occurred in either of them.

In the above solitary instance of this kind which has happened to M. Menière, a very careful examination of the body was made, for the purpose of ascertaining, if possible, the cause of the emphysema; but no satisfactory information could be obtained upon this point. In the upper part of the anterior mediastinum, there was a little air, but none in the lungs, which were insufflated, with every necessary precaution, to a state of distention, but no air escaped externally. The bronchia and trachea were uninjured: in fact, no appearance was detected which could furnish an elucidation of the phenomenon which had occurred during life.

In a consumptive patient, the two principal bifurcations of the bronchia were pressed upon by large tuberculous glands. The air introduced into the lung was frequently expelled during coughing with much violence; but the obstruction of the bronchia, which offered no opposition to the gradual admission of air, presented an effectual obstacle to its rapid exit: hence resulted violent dyspnœa, which was followed by a rupture of some of the pulmonary vesicles, and interlobular emphysema. The mediastinum was soon implicated, and the lower part of the neck was swollen during the last hour of life. In this instance, insufflation of the bronchial tubes considerably augmented the infiltration of air.

In two young chlorotic girls, who were subject to nervous disorders of all kinds, a hoarse and very loud cough gave rise to a swelling of the anterior part of the neck: it was thought that an obscure crepitation could be felt, but the fact was doubtful.*

The following case resembles the preceding as to its cause, but differs from them in the extension of the disease and its termination.

Case V. Joseph Mazau, æt. twenty, of very robust frame, was attacked, without any known cause, in the beginning of April 1826, with dyspnœa, which rapidly increased, and which was soon accompanied by an obstinate cough, with sanguineous expectoration: there was no fixed pain. Local and general bleeding procured relief. A few days after, he was attacked with slight colic and profuse diarrhœa: leeches were applied to the anus, with advantage. The man returned to his ordinary occupation, but he still continued weak, and unable to work for more than half a day at a time.

Complete convalescence appeared to be established, when he was again attacked, during the night, with dyspnœa and violent fits of coughing. In the midst of the efforts he involuntarily made, he felt his neck swell, but without experiencing any pain either in this part or the chest. The cough continued; the emphysema made very rapid progress; and the patient was admitted into the Hôtel Dieu, a few hours afterwards, in the following state: The neck was upon a level with the face; the whole surface of the body

* M. Breschet, in an interesting article on Emphysema, in the Dict. des Sc. Med., mentions the following case, which was communicated to him by M. Marjolin. A child, two and a half years old, was attacked with a violent convulsive cough, the paroxysms of which were very frequently repeated. On the morning of the fifth day, a few moments after a severe fit of coughing, an emphysematous swelling was seen above the sternum: the infiltration of air shortly extended, below the sterno-mastoid muscles, to the face, axilla, and the whole of the upper part of the chest. In the evening of the same day, the air had entered the cellular tissue on the back part of the neck, the abdominal parietes, and the scrotum. The following day the upper and lower limbs became emphysematous. The difficulty of breathing increased in proportion to the extension of the emphysema: there was imminent danger of suffocation, and laryngotomy was impracticable, on account of the enormous size of the neck, the integuments of which were on a plane with the face: the operation, indeed, would have been useless, as the opening of the aerial passage appeared to be situated in the substance of the lungs. By the advice of M. Dubois, the only treatment that was adopted consisted in the administration of narcotics, with pectoral drinks to calm the cough; the whole body was surrounded with compresses steeped in aromatic wine. Gradually the breathing became more free, the cough less frequent and severe, and in eight days the emphysematous affection spontaneously disappeared.—Editor.

was equally tumefied, and in every part a crepitation was distinct. The thorax was enormously swollen, and the arms puffed up to the wrists; cough slight, but frequent; eyes prominent; lips of a violet colour; pulse small and quick; patient in great alarm; abundance of viscid expectoration, with streaks of blood; general and copious perspiration; tongue red. The next day, a vertical incision, half an inch long, was made about the middle of the sternum: the air escaped with a slight hissing noise. During the whole of the day, the man occupied himself in pushing the air towards this opening, and towards evening nearly the whole of the chest had resumed its natural size.

On the third night after the opening had been made, he was attacked with delirium, fever, and great agitation. In the morning he was in a state of great depression: the emphysema had now extended to the knees; the wound in the chest had become gangrenous, without any very evident sign of previous inflammation. Blisters were applied to the thighs, and tonics were administered. The cerebral symptoms rapidly became worse, and death took place on the third day after the occurrence of delirium.

The body was examined twenty-five hours after death.

External appearances: General emphysema; no trace of decomposition; limbs flaccid.

Head: Pia mater remarkably thin, of a bright rose colour, entirely without any serous exudation, and adhering to the grey substance. This substance was of a similar colour, and upon its surface were numerous lenticular ecchymoses, of a deeper tint. The whole brain of a firm consistence.

Thorax: The whole of the sub-pleural cellular tissue filled with air, and there was a considerable quantity around the root of the lungs. The lungs themselves free from adhesions, and not emphysematous, but they were studded with miliary tubercles in a crude state. In the superior lobe of each lung were found small cavernous sinuses, formed by ulceration of the bronchial tubes. The mucous membrane of the trachea and bronchia of a reddish brown colour, very thick, and without any appearances of ulceration. The lungs were insufflated with great force, but not a single bubble of air was seen to escape.

Abdomen: Follicular ulceration in the small intestines. Mesenteric glands double their natural size, and soft in the centre. The subcutaneous cellular tissue was not altered in colour or consistence. For about an inch around the wound which had been made upon the sternum, it was of a reddish-brown colour, and condensed by a plastic matter, which had obstructed its permeability.

The circumstances under which the emphysema had been developed, left no doubt that it arose from the affection of the lungs. The air which filled the mediastinum and the cellular tissue which unites the pleura to the thoracic parietes, indicated its *point de départ;* but why did not the lungs participate in the general condition? There was no solution of continuity in the aerial passages; and, unless the ulcerated bronchial tubes had given passage to the air, its presence under the skin cannot be explained. But chronic ulceration of a tissue is always accompanied by a thickening of the surrounding cellular substances: besides which, insufflation proved that no air had escaped in that way. But little advantage was obtained by the exit given to the air: the thorax, it is true, was diminished in size, but even this amelioration was but temporary. The slight inflammation which took place in the wound rendered the cellular tissue impermeable, and the tumefaction quickly reappeared. The gangrene of the skin surrounding the wound arose from a similar cause with that which frequently attacks the punctures which are made in cases of anasarca. When the subcutaneous cellular tissue is distended, either with air or water, its vitality is diminished, and mortification ensues from but a trifling injury.*

---

### ITALIAN MEDICAL INSTITUTIONS AND PRACTICE.

* Breschet, in the article before referred to, in the Dict. des Sc. Medicales, quotes a curious fact from Fabricius Hildanus. In 1593, a child, eighteen months old, was exhibited in Paris, with a monstrous sized head. The parents of the unfortunate infant carried it from place to place, as an object of curiosity. Numbers flocked to see the wonder, but at length a sagacious magistrate suspected some deception: the parents were arrested, and confessed their crime, after being put to the rack. A small opening had been made upon the crown of the head, and air was blown under the skin by a tube: this operation was daily repeated until the head was of a sufficiently marvellous size. The parties were convicted, and condemned "a la peine capitale."—Editor.

